# Safety and Effectiveness of Tailored Hemorrhoidectomy in Outpatients Setting

**DOI:** 10.3389/fsurg.2021.708051

**Published:** 2021-08-16

**Authors:** Giovanni Tomasicchio, Gennaro Martines, Giuliano Lantone, Rigers Dibra, Giuseppe Trigiante, Michele De Fazio, Arcangelo Picciariello, Donato Francesco Altomare, Marcella Rinaldi

**Affiliations:** Surgical Unit “M. Rubino”, Department of Emergency and Organ Transplantation, University Aldo Moro of Bari, Bari, Italy

**Keywords:** hemorrhoids, ambulatory setting, milligan morgan hemorrhoidectomy, long term outcome, local anaestesia

## Abstract

**Introduction:** Single or double prolapsed pile instead of full muco-hemorrhoidal prolapse is a common finding in patients with symptomatic III or IV degree hemorrhoids. For this selected group of patients, relief of symptoms could be achieved by managing the single/double prolapsed piles instead of performing traditional hemorrhoidectomy. The aim of this single-center study was to evaluate the safety and medium- and long-term effectiveness of an outpatient tailored Milligan-Morgan hemorrhoidectomy (MMH) performed under local anesthesia (LA).

**Material and methods:** Clinical records of 202 patients submitted to outpatient tailored MMH, under LA and without anal dilation, treated between 2013 and 2020, were retrospectively reviewed using a prospectively maintained database and completed by a telephone interview or outpatient consultation. Postoperative pain score, the need for painkillers, postoperative complications and symptoms recurrence, return to working activities, and patient grading assessment scale were recorded.

**Results:** Thirty-five (17%) out of 202 patients recruited were lost to the follow-up. One hundred and fifty-two and 15 patients underwent a single and double pile hemorrhoidectomy, respectively. With regard to postoperative outcomes, visual analogue scale (VAS) decreased from a median value of 4 [interquartile range (IQR) 2–6] on the day of surgery to 1 (IQR 0–4) on the 10th postoperative day (*p* < 0.001). Sixty-one patients (37%) needed oral painkillers during the 1st week after surgery. There was no mortality or major postoperative complication. Bleeding requiring hospital readmission was reported in seven (4%) patients, and one patient underwent emergency surgery with no need for blood transfusion. No postoperative urinary retention, anal incontinence, or stricture occurred in the series. During the median follow-up of 39 (IQR 12–60) months, 26 patients (16%) reported symptoms of recurrence but only six underwent traditional MMH. Recovery to normal activity occurred within a median period of 6 days (IQR 3–10) and the Clinical Patient Grading Assessment Scale (CPGAS) at 1 year after surgery was reported to be a “good deal better.”

**Conclusions:** Tailored MMH performed under LA in an ambulatory setting can be considered a safe and effective technique with high compliance and satisfaction of patients.

## Introduction

Hemorrhoidal disease is one of the most common anorectal disorders, with an overall prevalence of 39% (1, 2). Conservative treatments are considered in the early stages, while surgery should be reserved for advanced grades or for the refractory of patients to conservative procedures (3–6). Among surgical procedures, the Milligan-Morgan hemorrhoidectomy (MMH) is still considered the “gold standard” for advanced grades of hemorrhoids (7, 8). However, this operation carries prolonged postoperative pain and convalescence and potential complications such as urinary retention, bleeding, and anal stricture (9). In the last decades, the use of new devices based on ultrasound or radiofrequency, such as Harmonic Scalpel® and Ligasure^TM^ system, has contributed to lower postoperative pain while shortening the recovery time (10–12).

Although, general or epidural anesthesia is the most commonly performed anesthetic techniques, local anesthesia (LA) is considered as a safe alternative with a significant reduction in complications (13–15). Ambulatory anorectal surgery is becoming a routine procedure for several proctologic diseases including fistula, abscess, condyloma, pilonidal disease, and hemorrhoids. The American Society of Colon and Rectal Surgeons suggests that 90% of anorectal diseases might be suitable for ambulatory surgery, with consequent reduction in hospital admissions and hospital charges (16).

However, several patients affected by symptomatic III or IV grade hemorrhoids, with a single or double prolapsed pile, could benefit from a limited excision under LA performed in the ambulatory setting without the need for anal dilation. For this selected group of patients, traditional hemorrhoidectomy could result in an overtreatment even if the recurrence of hemorrhoidal prolapse in other quadrant may occur several years later (17).

This retrospective single-center study aimed is to evaluate the safety and long-term effectiveness of outpatient MMH performed without anal dilation under LA in patients with single or double pile hemorrhoids.

## Materials and Methods

A retrospective observational study was carried out using a prospectively maintained database of patients who underwent outpatient MMH in a tertiary colorectal unit between September 2013 and January 2020. Follow-up data were collected by a telephone interview or further outpatient consultations. Consecutive patients over 18 years old, with symptomatic grade III-IV hemorrhoids according to the classification of Goligher involving a single or double external piles, that fit for operation under LA (ASA I/II), were enrolled in the study.

Exclusion criteria were the use of anticoagulants or immunosuppressive drugs, pregnancy, severe constipation, concomitant anal condition requiring surgical treatment, previous anal operations for anal fissure or fistula, patients living too far (more than 30 min driving) from the hospital, and allergy to anesthetic drugs.

Postoperative pain at 30 min, 5 and 10 days after surgery was evaluated using a VAS. Postoperative complications, the number of painkillers used, and days to return to normal activity were recorded.

Postoperative clinical outcomes (bleeding and recurrence) and satisfaction of the patient, scored by the Clinical Patient Grading Assessment Scale (CPGAS) (18), were evaluated after a minimum period of 1 year of follow-up.

## Surgical Procedure

After obtaining written informed consent, all patients were placed in prone or Sims position and received LA by injecting mepivacaine hydrochloride 20 mg/ml, in the submucosa of the prolapsed pile. Harmonic Scalpel® (ETHICON ENDO-SURGERY, LLC, Guaynabo, PR, USA) was used to remove the prolapsed hemorrhoidal piles without anal dilation. The terminal hemorrhoidal artery was just coagulated without ligation. The power of the Harmonic Scalpel® was set at level 3. A resorbable hemostatic swab, made by oxidized regenerated cellulose, was applied into the anal canal at the end of the procedure. Application of hemostatic stitches was considered only in case of incomplete hemostasis.

Patients were revaluated 30 min after the procedure to verify the achieved hemostasis and discharged immediately and reevaluated at 5 and 10 days.

**Figure 1:**
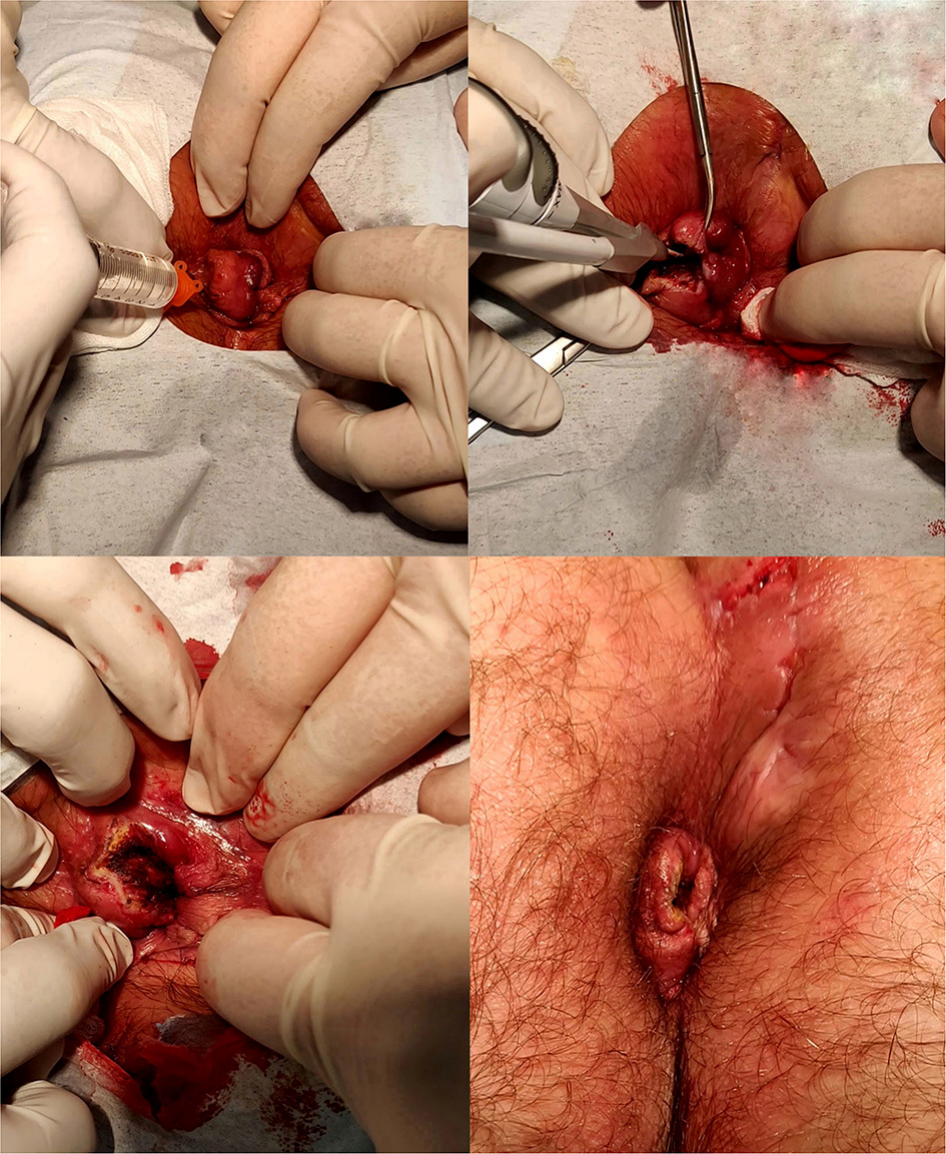
Main steps of the surgical procedure.

Bulking stool softeners (Psyllogel Nathura® s.p.a., Montecchio Emilia, RE, Italia) were prescribed irrespectively of the bowel habit for 1 month. Painkillers (Ketorolac 10 mg or paracetamol 1,000 mg pills) were taken in case of anal pain. No antibiotic prophylaxis was prescribed. The procedure was performed by resident doctors under the supervision of board-certified colorectal surgeons (Figure 1).

## Statistical Analysis

All data were expressed as median range and interquartile (IQR), and the statistical analysis to compare the changes in VAS at different times was performed using paired Wilcoxon rank-sum test. A value of *p* < 0.05 was considered statistically significant. Descriptive data were expressed as percentage. Statistical analysis was carried out using RStudio [R version 4.0.3 Copyright (C) 2020 The R Foundation for Statistical Computing].

## Results

Two hundred and twelve patients (median age 54.57 IQR 45–65, women 51%) with 1 or 2 symptomatic III or IV degree piles entered in the study. One hundred and sixty-seven patients (women 51%) agreed to participate in the telephone interview or were controlled after a median follow-up of 39.1 (IQR 12–60) months, while the remaining 35 (17%) were lost to the follow-up.

There were 82 men (49%) and 85 women (51%) with a median of 53 years (IQR 45–64) and 55 years (IQR 46–64), respectively. Twenty-five (17%) patients have had previous hemorrhoidal surgical treatments. Eight patients were treated by MM technique, seven by rubber band ligation, seven by stapler device, and three patients by trans-anal Doppler-guided hemorrhoid artery ligation.

One hundred and forty-seven patients (87%) were affected by a grade III and 21 patients (13%) by grade IV hemorrhoids. One hundred and fifty-two (91%) patients underwent a single pile removal, while 15 (9%) had the removal of two piles with a median operative time of 10 min.

**Figure 2:**
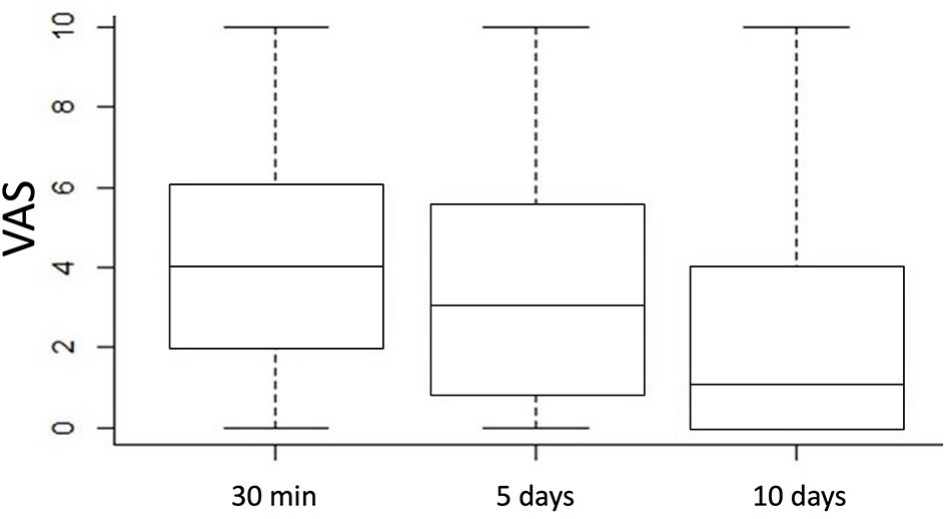
Postoperative pain according to VAS 30 min, 5 and 10 days after surgery.

The 30-min postoperative pain had a median VAS of 4 (IQR 2–6), which decreased to 3 (IQR 1–5.5) (*p* = 0.007161) 5 days later and to 1 (IQR 0–4) (*p* < 0.001) 10 days later (Figure 2).

No significant difference in terms of pain was found comparing single and double pile removal (Table 1).

**Table 1:** Evaluation of pain after single and double pile removal.

	**VAS 30 min**	**VAS 5 days**	**VAS 10 days**
Single Pile*n = 151*	4 (IQR 2–6)	3(IQR 1–5)	1(IQR 0–4)
Double Piles*n = 16*	4 (IQR 2–5)	2(IQR 1–4)	2(IQR 0.75–2.25)
*p-value*	0.87	0.84	0.43

One hundred and six patients (63%) did not use painkillers during the postoperative period, while 61 (37%) needed paracetamol or ketorolac administration during the first 1st after surgery. Four patients (3%) were reevaluated within 1 month because of persisting anal pain.

No correlation was found between age, previous surgery, sex, and the severity of the pain.

No mortality or major postoperative complications were recorded. Minor bleeding was reported by 43 patients (26%) within the first 10 postoperative days, with spontaneous resolution. Bleeding requiring hospital readmission was reported in seven patients (4%), but only one (0.6%) developed significant anemia (Hb level 7 g/dl) requiring surgery without the need of blood transfusion, while six had conservative treatment and were discharged the day after admission.

Wound infection was reported in two cases (1%). No patient had postoperative urinary retention neither had anal incontinence or stricture.

During the follow-up period, 26 patients (16%) developed some degree of prolapse recurrence, but only 6 (4%) of them underwent a new surgical treatment (traditional MM), while the remaining 20 patients (12%) were treated conservatively (Table 2).

**Table 2:** Postoperative complications and recurrence rates.

	***n* = 167**
Follow-up (months)	39.1 (12–60)
**Post Bleeding**	
No	117 (70%)
Yes	50
- Minor	43 (26%)
- Major	7 (4%)
**Complications**	
No	161 (96%)
Yes	6
- Pain	4 (3%)
- Wound infection	2 (1%)
**Recurrence**	
No	141 (84%)
Yes	26 (16%)
- Surgery	
No	20 (12%)
Yes	6 (4%)

Patients were able to recover their normal activity and work after a median period of 6 days (IQR 3–10), and the CPGAS at 1 year after surgery was a “good deal better” with a median value of 5 (IQR 4–6).

## Discussions

Milligan-Morgan operation is traditionally performed in the operating theater under general or epidural anesthesia. However, in the last few decades, there has been an increasing trend to perform anal surgery in an ambulatory setting under LA with or without intravenous sedation. The American Society of Colon and Rectal Surgeons recommends to consider ambulatory surgery in most patients whenever proctological procedures are contemplated. In fact, this surgical approach has been shown to be safe and effective with reduction in the hospital charges and with high satisfaction of patients (16).

This study demonstrates that a selected group of patients with single or double III/IV grade hemorrhoids can be treated safely in an ambulatory setting by a tailored MM operation using ultrasound devices under LA without the need for anal dilation.

In fact, in this study, postoperative complications occurred in <10 % of the patients. Seven patients (4.2%) reported major bleeding requiring hospital readmission, but only one needed a new surgical treatment, while the remaining six were managed conservatively.

The absence of anal dilatation reduces complications while increasing compliance of patients; in fact, anal dilatation has been shown to potentially cause anal sphincter fragmentation leading to fecal incontinence in some patients (19).

This surgical option can be considered effective in the medium/long-term outcome since a symptomatic recurrence was recorded in only 16% of them after a median of 3 years (IQR 2–5) follow-up, and only 4% needed a standard MM operation.

Several studies report long-term results after MM operation (20–22); however, no study reports the effectiveness of MMH performed under LA without anal dilation in an ambulatory setting in terms of recurrence rate.

Furthermore, the subjective overall evaluation of the results of surgery using the CPGAS indicated that the patients were “a good deal better” after surgery with a return to normal activity and work within 1 week.

Several meta-analyses demonstrated that MMH performed under LA is associated with significantly lower postoperative pain within 24 h after surgery and a lower need for painkiller drugs compared to general or spinal anesthesia (13, 14).

In agreement with the literature, our study reported that these patients complained only a “troublesome pain” (median VAS 4) in the early postoperative time, with a reduction to a median value of 3 on the 5th postoperative day. Furthermore, although, Haveran et al. (23) suggest that the maximum benefit can be realized by the association of LA with propofol/ketamine intravenous sedation, in our series, no intravenous sedation was needed.

Further contribution to minimize the postoperative pain probably results from the use of the Harmonic Scalpel® that has been demonstrated to lower the postoperative pain compared to the diathermy, due to the little spread of the thermal injury, and by avoiding hemostatic suture to the terminal hemorrhoidal artery (24–26).

In our experience, 37% of the patients needed painkillers in the 1st week after surgery and none of them used opioids, while only four of them reported pain at defecation after 1 month. These patients were treated by analgesic and use of bulk stool softeners for further 2–3 weeks.

Despite urinary retention complicates up to 50% of patients undergoing anorectal surgery under spinal anesthesia (27, 28), particularly those undergoing hemorrhoidectomy (29), in our series no cases of urinary retention were recorded. The absence of episodes of urinary retention in this study may be related to the use of LA; in fact, Xia et al. (13), in their meta-analysis, reported a significantly reduced risk of urinary retention after the procedure performed under LA compared to general or spinal anesthesia.

One possible disadvantage of LA is the fear of pain during the anesthetic injection, which can be minimized by the local application of anesthetic ointments before the injection (14).

Postoperative bleeding is another common complication requiring reoperation. The rate of minor bleeding, in our series, was 26%, but all the patients had a spontaneous resolution in the first 10 days. Only 4% of our patients had major bleeding requiring hospitalization; however, only one patient required surgery without the need of blood transfusion. These data match positively with a reported rate of delayed posthemorrhoidectomy bleeding in the literature, which is about 5% (30). The use of the Harmonic Scalpel® contributes to achieving safe hemostasis while minimizing the thermal injury of the surrounding tissues, allowing faster wound healing (10, 23).

The surgical option to treat these patients by a minimal tailored approach got a high grade of patient satisfaction, not only because of the advantages of the ambulatory setting (short duration of the procedure, rapid return home, and minimal off-work period) but also because of the comfortable prone or Sims position without the need for anal dilatation and for absence of intravenous sedation. In fact, the subjective overall evaluation of the results of this surgery using the CPGAS score indicated that the patients were “a good deal better.”

The main limitation of this study is its retrospective nature, and the follow-up data have been collected only by a telephone interview although, those complaining of symptom recurrence were controlled as outpatients.

## Conclusions

Tailored ambulatory MMH under LA without anal dilation can be considered a safe and effective technique for patients affected by single or double III/IV grade hemorrhoids with high compliance and satisfaction of patients.

## Data Availability Statement

The raw data supporting the conclusions of this article will be made available by the authors, without undue reservation.

## Ethics Statement

The studies involving human participants were reviewed and approved by Comitato Etico Indipendente - Azienda Ospedaliera Universitaria Policlinico di Bari. Written informed consent for participation was not required for this study in accordance with the national legislation and the institutional requirements.

## Author Contributions

GTo and AP: conception and design of the study, acquisition analysis and interpretation of data, writing the paper, and final approval of the version to be published. GM, GL, RD, GTr, MD, and MR: acquisition analysis and interpretation of the data and final approval of the version to be published. DA: conception and design of the study, interpretation of the results, writing the paper, and final approval of the version to be published. All authors contributed to the article and approved the submitted version.

## Conflict of Interest

The authors declare that the research was conducted in the absence of any commercial or financial relationships that could be construed as a potential conflict of interest.

## Publisher's Note

All claims expressed in this article are solely those of the authors and do not necessarily represent those of their affiliated organizations, or those of the publisher, the editors and the reviewers. Any product that may be evaluated in this article, or claim that may be made by its manufacturer, is not guaranteed or endorsed by the publisher.

## References

[1] Riss S Weiser FA Schwameis K Riss T Mittlbock M Steiner G. The prevalence of hemorrhoids in adults. Int J Colorectal Dis. (2012) 27:215–20. 10.1007/s00384-011-1316-3 21932016

[2] Gallo G Sacco R Sammarco G. Epidemiology of hemorrhoidal disease. In: Ratto C Parello A Litta F editors. Hemorrhoids. Cham: Springer International Publishing. (2017) p. 1–5. 10.1007/978-3-319-51989-0_1-1

[3] Cocorullo G Tutino R Falco N Licari L Orlando G Fontana T. The non-surgical management for hemorrhoidal disease. A systematic review. G Chir. (2017) 38:5–14. 10.11138/gchir/2017.38.1.005 28460197 PMC5730401

[4] Picciariello A Tsarkov PV Papagni V Efetov S Markaryan DR Tulina I. Classifications and clinical assessment of haemorrhoids: the proctologist's corner. Rev Recent Clin Trials. (2021) 16:10–6. 10.2174/1574887115666200312163940 32164517

[5] Lobascio P Laforgia R Novelli E Perrone F Di Salvo M Pezzolla A. Short-term results of sclerotherapy with 3% polidocanol foam for symptomatic second- and third-degree hemorrhoidal disease. J Invest Surg. (2020). 10.1080/08941939.2020.1745964. [Epub ahead of print]. 32290709

[6] Gallo G Martellucci J Sturiale A Clerico G Milito G Marino F. Consensus statement of the Italian society of colorectal surgery (SICCR): management and treatment of hemorrhoidal disease. Tech Coloproctol. (2020) 24:145–64. 10.1007/s10151-020-02149-1 31993837 PMC7005095

[7] van Tol RR Kleijnen J Watson AJM Jongen J Altomare DF Qvist N. European Society of ColoProctology: guideline for haemorrhoidal disease. Colorectal Dis. (2020) 22:650–62. 10.1111/codi.14975 32067353

[8] Altomare DF Picciariello A Pecorella G Milito G Naldini G Amato A. Surgical management of haemorrhoids: an Italian survey of over 32 000 patients over 17 years. Colorectal Dis. (2018) 20:1117–24. 10.1111/codi.14339 30004171

[9] Watson AJ Hudson J Wood J Kilonzo M Brown SR McDonald A. Comparison of stapled haemorrhoidopexy with traditional excisional surgery for haemorrhoidal disease (eTHoS): a pragmatic, multicentre, randomised controlled trial. Lancet. (2016) 388:2375–85. 10.1016/S0140-6736(16)31803-7 27726951 PMC5269572

[10] Lim DR Cho DH Lee JH Moon JH. Comparison of a hemorrhoidectomy with ultrasonic scalpel versus a conventional hemorrhoidectomy. Ann Coloproctol. (2016) 32:111–6. 10.3393/ac.2016.32.3.111 27437393 PMC4942526

[11] Jayne DG Botterill I Ambrose NS Brennan TG Guillou PJ O'Riordain DS. Randomized clinical trial of Ligasure versus conventional diathermy for day-case haemorrhoidectomy. Br J Surg. (2002) 89:428–32. 10.1046/j.0007-1323.2002.02056.x 11952582

[12] Gallo G Realis Luc A Clerico G Trompetto M. Diathermy excisional haemorrhoidectomy - still the gold standard - a video vignette. Colorectal Dis. (2018) 20:1154–6. 10.1111/codi.14430 30298969

[13] Xia W MacFater HS MacFater WS Otutaha BF Barazanchi AWH Sammour T. Local anaesthesia alone versus regional or general anaesthesia in excisional haemorrhoidectomy: a systematic review and meta-analysis. World J Surg. (2020) 44:3119–29. 10.1007/s00268-020-05555-6 32383052

[14] Mohamedahmed AYY Stonelake S Mohammed SSS Zaman S Ahmed H Albarade M. Haemorrhoidectomy under local anaesthesia versus spinal anaesthesia: a systematic review and meta-analysis. Int J Colorectal Dis. (2020) 35:2171–83. 10.1007/s00384-020-03733-5 32862302

[15] Luo CH Zang CB Zhang GK Liu HY. Haemorrhoidectomy by vessel sealing system under local anaesthesia in an outpatient setting: preliminary experience. Colorectal Dis. (2010) 12:236–40. 10.1111/j.1463-1318.2009.01833.x 19508547

[16] Ternent CA Fleming F Welton ML Buie WD Steele S Rafferty J. Clinical practice guideline for ambulatory anorectal surgery. Dis Colon Rectum. (2015) 58:915–22. 10.1097/DCR.0000000000000451 26347962

[17] Elbetti C Giani I Novelli E Martellucci J Feroci F. Symptomatic pile tailored procedure. A new perspective for hemorrhoidal disease treatment. Ann Ital Chir. (2017) 88:348–51. 29051401

[18] Heckert J Sankineni A Hughes WB Harbison S Parkman H. Gastric electric stimulation for refractory gastroparesis: a prospective analysis of 151 patients at a single center. Dig Dis Sci. (2016) 61:168–75. 10.1007/s10620-015-3837-z 26280084

[19] Speakman CT Burnett SJ Kamm MA Bartram CI. Sphincter injury after anal dilatation demonstrated by anal endosonography. Br J Surg. (1991) 78:1429–30. 10.1002/bjs.1800781206 1773315

[20] Ganio E Altomare DF Milito G Gabrielli F Canuti S. Long-term outcome of a multicentre randomized clinical trial of stapled haemorrhoidopexy versus Milligan-Morgan haemorrhoidectomy. Br J Surg. (2007) 94:1033–7. 10.1002/bjs.5677 17520710

[21] Johannsson HO Graf W Pahlman L. Long-term results of haemorrhoidectomy. Eur J Surg. (2002) 168:485–9. 10.1080/110241502321116505 12549690

[22] Mattana C Coco C Manno A Verbo A Rizzo G Petito L. Stapled hemorrhoidopexy and Milligan Morgan hemorrhoidectomy in the cure of fourth-degree hemorrhoids: long-term evaluation and clinical results. Dis Colon Rectum. (2007) 50:1770–5. 10.1007/s10350-007-0294-6 17701371

[23] Haveran LA Sturrock PR Sun MY McDade J Singla S Paterson CA. Simple harmonic scalpel hemorrhoidectomy utilizing local anesthesia combined with intravenous sedation: a safe and rapid alternative to conventional hemorrhoidectomy. Int J Colorectal Dis. (2007) 22:801–6. 10.1007/s00384-006-0242-2 17119982

[24] Talha A Bessa S Abdel Wahab M. Ligasure, Harmonic Scalpel versus conventional diathermy in excisional haemorrhoidectomy: a randomized controlled trial. ANZ J Surg. (2017) 87:252–6. 10.1111/ans.12838 25214362

[25] Abo-hashem AA Sarhan A Aly AM. Harmonic Scalpel compared with bipolar electro-cautery hemorrhoidectomy: a randomized controlled trial. Int J Surg. (2010) 8:243–7. 10.1016/j.ijsu.2010.01.010 20132916

[26] Khan S Pawlak SE Eggenberger JC Lee CS Szilagy EJ Wu JS. Surgical treatment of hemorrhoids: prospective, randomized trial comparing closed excisional hemorrhoidectomy and the Harmonic Scalpel technique of excisional hemorrhoidectomy. Dis Colon Rectum. (2001) 44:845–9. 10.1007/BF02234706 11391146

[27] Choi S Mahon P Awad IT. Neuraxial anesthesia and bladder dysfunction in the perioperative period: a systematic review. Can J Anaesth. (2012) 59:681–703. 10.1007/s12630-012-9717-5 22535232

[28] Prasad ML Abcarian H. Urinary retention following operations for benign anorectal diseases. Dis Colon Rectum. (1978) 21:490–2. 10.1007/BF02586733 710240

[29] Zaheer S Reilly WT Pemberton JH Ilstrup D. Urinary retention after operations for benign anorectal diseases. Dis Colon Rectum. (1998) 41:696–704. 10.1007/BF02236255 9645737

[30] Jeong HY Hwang DY Cho DH Lee JK. Analysis of risk factors for delayed bleeding after semi-closed hemorrhoidectomy. Int J Colorectal Dis. (2021) 36:857–64. 10.1007/s00384-021-03895-w 33661360

